# Development and Validation of Vitamin D- Food Frequency Questionnaire for Moroccan Women of Reproductive Age: Use of the Sun Exposure Score and the Method of Triad’s Model

**DOI:** 10.3390/nu15040796

**Published:** 2023-02-04

**Authors:** Noura Zouine, Ilham Lhilali, Aziza Menouni, Lode Godderis, Adil El Midaoui, Samir El Jaafari, Younes Zegzouti Filali

**Affiliations:** 1 Cluster of Competency “Environment and Health”, Faculty of Sciences, Moulay Ismail University, Meknes 50000, Morocco; 2 Higher Institute of Nursing and Health Professions of Fes-Meknes Annex, Meknes 50000, Morocco; 3 Health and Environment Unit, Faculty of Medicine, KU Leuven, 3000 Leuven, Belgium; 4 IDEWE, External Service for Prevention and Protection at Work, 3001 Heverlee, Belgium; 5 Faculty of Sciences and Techniques, Errachidia, Moulay Ismail University of Meknes, Errachidia 52000, Morocco; 6 Department of Pharmacology and Physiology, Faculty of Medicine, University of Montreal, Montreal, QC H3C 3J7, Canada; 7 BASE Laboratory, FSM-FSTE, Moulay Ismail University of Meknes, Meknes 50000, Morocco

**Keywords:** vitamin D, food frequency questionnaire, women of reproductive age, method of triads, hypovitaminosis D, dietary assessment, sun exposure score

## Abstract

This cross-sectional study aimed to develop and validate a vitamin D food frequency questionnaire (VitD-FFQ) to assess vitamin D intake in Moroccan women of reproductive age. Using the method of triads, the VitD-FFQ was validated against seven-day dietary records (7d-FR) and 25-hydroxyvitamin D (25(OH)D) as a biomarker of vitamin D status in 152 women (aged 18–45 years). Participants’ sun exposure scores (SES) were assessed using a specific questionnaire (SEQ). Predictors of vitamin D status were identified via linear regression models. Several statistical tests were applied to evaluate the criterion validity of the FFQ against two references methods (7d-FR and the biomarker-serum 25(OH)D). Median (Interquartile range) intakes were 7.10 ± 6.95 µg /day and 6.33 ± 5.02 µg/ day, respectively, for VitD-FFQ and 7d-FR. Vitamin D status was mainly determined by SES (R = 0.47) and vitamin D absolute food intakes derived by the VitD-FFQ (R = 0.56), which demonstrated a more significant prediction ability compared to 7d-FR (R = 0.36). An agreement was observed between the VitD-FFQ and 7d-FR (BA index of 3.29%) with no proportional bias (R^2^ = 0.002, *p* = 0.54). <10% of participants were incorrectly classified, and weighted kappa statistics showed that VitD-FFQ had an acceptable ranking ability compared to the 7d-FR and the biomarker. The validity coefficient for the VitD-FFQ was high: ρQR = 0.90 (95%CI: 0.89–0.92), and a range from 0.46 to 0.90. Adjustment for the participants’ SES and BMI (body mass index) improved the biomarker’s validity coefficient (ρRB 0.63 (95% CI 0.39–0.82). Our results indicate that the VitD-FFQ is valid for estimating absolute vitamin D intake in Moroccan women of reproductive age.

## 1. Introduction

Althouμgh biologically inactive, vitamin D is an essential nutrient that can be converted to the steroid hormone 1,25(OH)2D3 and influences multiple processes unrelated to calcium metabolism while regulating gene transcription [1]. Over the past decades, extensive research revealed significant associations between vitamin D status and various health outcomes [2,3]. Studies have reported that vitamin D deficiency is associated with skeletal disorders (i.e., rickets in children, osteoporosis and osteomalacia in adults) [4], chronic disorders, such as diabetes type I mellitus [5], respiratory infections and asthma [6], cardiovascular diseases and hypertension [7,8], and cancers [8,9]. Large–scale observational studies support the crucial role of vitamin D in women’s reproduction and offspring development [9,10,11]. More recently, vitamin D deficiency has emerged as a possible contributor to the cytokine storm that heralds some of the most severe COVID-19 disease complications [12,13].

Sun exposure is the primary determinant of vitamin D status during more than 99% of human evolution. Vitamin D3 (Cholecalciferol) might be generated endogenously when the epidermis is exposed to sunlight [4,14]. The amount of vitamin D3 synthesized in this process is affected by numerous factors, including latitude, seasons, time of the day, skin pigmentation, air pollution, clothing, and sunblock lotions use [15]. Thus, to have sufficient vitamin D3 production, certain researchers advise sun exposure to the face, arms, hands, and legs for around 5 to 30 min, preferably between 10 a.m. and 4 p.m., daily or at least twice per week without the use of sunscreen [16,17].The second and exogenous source of vitamin D3 is a diet, such as animal sources and plants (in the form of ergocalciferol (D2)) [16]. Notably, food sources containing significant amounts of this nutrient are very scarce, such as oily fish (i.e., salmon, sardines and tuna) and oils of cod liver, beef liver, yolk, and sun-exposed mushrooms [3,16,18].Specific orange juice formulations, yoghurt, cheese, margarine, bread, and breakfast cereal may also be fortified with vitamin D [19].The serum level of 25(OH)D indicates the overall synthesis of vitamin D from both dietary and solar sources [20]. Nevertheless, under conditions where UVB exposure is limited, vitamin D production in the skin may be altered in contrast, and this nutrient must be supplemented in the diet regularly [14,21]. Depending on the country, guidelines for the recommended supplemented dose vary from 200 IU to 2000 IU daily in children and adults [17].

It is noteworthy that, dietary intake measures are challenging, and no single method can accurately estimate dietary exposure [22]. Food frequency questionnaires (FFQs) are self-report methods used in large-scale epidemiological research to examine long-term dietary intakes. It is a reasonably simple, inexpensive, and time-efficient [22,23]. Even in the case of a nutrient with restricted dietary sources such as vitamin D, FFQs are very effective [24]. It can estimate usual intake and classify individual intakes into broad categories to allow meaningful comparisons of the attributes associated with high or low intakes [25]. However, FFQ should be developed specifically for each study group and research purposes because diet may be influenced by various factors, including ethnicity, culture, individual’s preferences, and economic status [26,27]. Prior validation of the instrument is also a fundamental step, since incorrect information can lead to false associations between diet and the onset of certain diseases [28].

In the validation process, the FFQ is compared to other self-report methods as reference methods, such as 24 h-recalls or food records [29]. However, both the instrument being tested (FFQ) and the reference method present similar random and systematic errors due to reliance on memory as well as inaccuracies related to the estimation of reported food consumption [29,30]. These random errors cannot be predicted and may result in more significant correlation estimations between the two methods, thereby the FFQ’s validity [30]. Using nutritional biomarkers as an additional reference method in a triangle validation technic may improve validity estimates since measurement errors in biomarkers are essentially uncorrelated with errors in any dietary assessment [29,31]. Ultimately, the method of triads is widely used to provide a more objective metric in validation studies of vitamin D FFQs [32,33,34,35].

Likewise, based on Koppen Climate Classification, Morocco has “dry-summer subtropical” climates, which are often referred to as “Mediterranean”, with a burst of year-round sunshine [36]. Nevertheless, hypovitaminosis is emerging as a major public health concern. Studies show a high prevalence of hypovitaminosis D in the general female population [37]. This prevalence ranges between 78.1 and 98.4% when the 25OHD threshold is defined at concentrations <20 ng/mL, while it affects 90% of women with levels <30 ng/mL [37].

Numerous factors are related to this prevalence such as cultural aspects and lifestyle, which discourage women from spending time in the sun (i.e., wearing protective or restricting clothes and staying at home) [37,38,39,40], and additionally contribute to the widespread obesity and dark skin in many parts of the country [37]. Under such conditions, vitamin D status relies greatly on dietary sources, which might not be enouμgh to meet the requirements. Moroccans’ dietary habits are believed to follow a Mediterranean pattern, generally characterized by low to moderate consumption of cheese, yoghurt, fish, poultry and eggs and low consumption of red meat, which are all vitamin D-rich foods [41]. The government mandated a fortification program in the nation, allowing less than 300 IU of vitamin D3 in the daily ration of some food products [42]. However, there is scant accurate information on vitamin D habitual dietary intake among the Moroccan population. To our best knowledge, no specific questionnaire has been developed or validated to determine the daily intake and frequency of consumption of vitamin D-rich/fortified foods.

Therefore, the main objective of this study was to develop a new self-administrated FFQ to estimate the daily intake of vitamin D in Moroccan women of reproductive age (18–45 years) and to assess the criterion validity of the FFQ using the method of triads. The vitamin D dietary intake estimated by the VitD-FFQ was compared with the daily intake measured by 7d-FR and with the 25(OH) D serum concentrations. We applied the SEQ and the VitD-FFQ simultaneously (with respect to the known half-life of circulating 25(OH)D) to enable accurate estimate of the validity coefficient of the FFQ while controlling for endogenously synthesis induced by the sun exposure. Comparison of the dietary assessment tools by their ability to explain the objective measures of the nutrient was also performed.

## 2. Materials and Methods

### 2.1. Study Design, Population Enrollment and Samples Size

**Figure 1:**
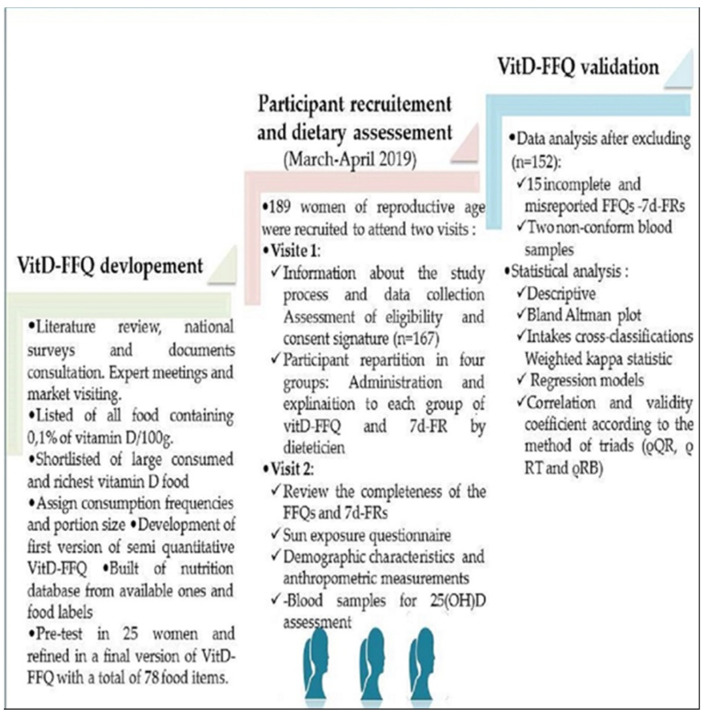
The development and validation process algorithm of the VitD-FFQ.

This was a cross-sectional study in which the vitamin D intake of participants was estimated using the method of triads that combine three assessment methods: FFQ, 7d-FR and vitamin D serum biomarker. The study process is represented in Figure 1. Healthy women of reproductive age (18–45 years) living in Meknes province and surrounding areas were recruited throuμgh an announcement at Moulay Ismail university population. The latitude of the chosen study area is around 33.89° which make vitamin D skin synthesis possible across 300/364 days of the year [43]. Therefore, participant recruitment was performed between March and April 2019, in early spring season, when UVB strength would probably be moderate.

The literature identifies a sample size of 100 individuals as a quality criterion for validation studies [44]. A minimum of 50 subjects is indicated when using biomarkers as a reference method [44,45]. Similarly, for applying the Bland Altman approach, at least 50 is preferable to evaluate the limits of agreement [28]. Since 189 participants replied to our invitation voluntarily and were enrolled in the present study, the sample size was then considered as satisfactory. Exclusion criteria for the study were: women involved in loss weight programs, diet restriction (i.e., veganism or vegetarianism, lactose intolerance), or under medication known to interfere with vitamin D metabolism, such as glucocorticoids, carbamazepine used to treat epilepsy or Cholesterol-lowering druμgs, statin [46]. Furthermore, pregnant or breast-feeding women and women with self-reported medical conditions were excluded (i.e., severe anemia, hemophilic syndrome and malabsorption syndromes that may cause decreased vitamin D) [47].

All participants were invited to attend two appointments for data collection.

In visit 1: All participants were informed about the study process and the data collection. Next, inclusion/exclusion criteria were verified. Screening identified 167 women of reproductive age eligible and agreed to participate in the study. Afterwards, participants were divided into four groups and a dietitian explained and administered the ViD-FFQ and 7d-FR to each group. However, food record brochures that included written instructions and examples were provided individually to complete at home. The participants were asked to complete the VitD-FFQ and then the 7d-FR the following week to avoid memory bias between the dietary assessments. In order to reduce the social desirability bias, it was clearly explained to all participants that the aim of the study was nothing more than the evaluation of the VitD-FFQ as opposed to their food habits.

In visit 2: One week later, another visit was organized, and all VitD-FFQs and the 7d-FR were reviewed upon completion and submission by the same dietitian as the first visit. We only included the participants who fully completed and returned their 7d-FR. Incomplete FFQs and FR were excluded (i.e., missing items or day, an unreported quantity of food product and portion sizes consumed and others). In addition, blood samples and anthropometric measurements were taken by two registered nurses. All participants were interviewed to evaluate their sun exposure score during the last month using the sun exposure questionnaire (SEQ). The demographic information, such as the age and socioeconomic status of all participants, was also collected using a general questionnaire.

A total of 154 VitD-FFQs and the associated 7-FRs were of good quality of completeness. However, two participants failed to provide a blood sample. Therefore, the total number of women included in the analysis was 152. The ethics committee of biomedical research at Moulay Ismail University (reference; N°1/CERB-UMI/19) approved the study protocol, and all investigations were conducted under the principles of the Declaration of Helsinki. All participants signed written informed consent.

### 2.2. VitD-FFQ Description and Development

Different approaches were employed to develop the FFQ: Data from national population surveys and published scientific literature were compiled to inventory the dietary habits and foods commonly consumed by the Moroccan population [48,49,50,51]. Visits to typical supermarkets were made to identify local foods fortified with vitamin D3, their consequent brands and packaging labels. Subsequently, a list of foods naturally rich in vitamin D (at least 0.1 ng/100 g food) and supplemented was established [42,52,53,54,55]. In sum, 78 food items were incorporated in the final version of the VitD-FFQ. The foods were grouped into eight categories: Dairy products and beverages, eggs, fish and seafood, meat and products, fatty products (butter, margarine and oil), breakfast cereals, bakery and Moroccan biscuits, chocolate and cocoa.

The concept for the VitD-FFQ was based on a Belgium food-frequency questionnaire developed to assess the usual intake of methyl-group donors in women [56]. For each food item in the VitD-FFQ was assigned 3–5 daily portion size categories and a list of standard measures referring to commonly used household utensils (e.g., plates, glass, bowl, spoons) or “natural” units (e.g., one egg). Photographs from a booklet of Food and typical preparations of the Moroccan population’ were also showed to the participant as examples of portion size [57]. In the case of particular commercial fortified food, we incorporate the portion on product packaging labels (e.g., breakfast cereals). The developed vitamin D FFQ had to reflect the food consumption of the last month as we consider the 15-day half-life of 25(OH)D in circulation [58]. Therefore, participants were asked to indicate whether they had consumed the selected food items in the previous month in increasing six frequencies (monthly and weekly basis): never or less than once a month; 1–3 d/month; 1 d/week; 2–4 d/week; 5–6 d/week; every day [59]. Two open questions were asked at the end of the questionnaire to address the usual participant consumption of foods that were not included in the FFQ (frequency, brand/type and quantity) in addition to any nutritional supplements of vitamin D during the last month (type, dosage and frequency of dietary supplements).

The amounts of food consumed per day were computed by multiplying the averages of the frequencies taken as a standard unit (i.e., one serving/week = 0.14 serving/day) with the mean of the serving size range that the participants reported [56]. As a result, the daily vitamin D intakes were calculated by multiplying the quantities of Food consumed per day by the value of the vitamin D content per 100 g of product.

As no Moroccan food composition database that provides the food amount values of vitamin D is available, the nutritional content was derived from the most recent versions of the French CIQUAl food composition database [55]. The Nutrient Database for Dietary Studies 2015–2016 of the United States Department of Agriculture National Food (USDA) [60] was used when values were missing in the previously cited database.

Finally, in order to obtain the most reliable estimate of intake, the vitamin D content value for local and fortified Food (e.g., breakfast cereals and oils) were investigated to brand level detail and packages’ labeling. When necessary, we contacted companies to obtain the nutrient values of their product. A nutrient calculation tool was built in-house to evaluate vitamin D intake derived from the FFQs using Microsoft ^®^ Excel^®^ 2013.

Before starting the validation study, the FFQ was pre-tested to address content and comprehension-related issues, in a convenience sample (*n* = 25) who did not participate in the main study. The timing to answer the developed FFQ took approximately 20 to 30 min.

### 2.3. Description of the 7d-FR

We choose to incorporate vitamin D dietary intake from 7d-FR as a first reference technique in the method of triads in order to collect accurate quantitative information on individual food consumed during the validation period [59]. The food record, in contrast with the FFQ, is open-ended, does not rely on memory, and includes a direct estimate of portion size [28].

Moreover, with seven consecutive recording days of the week, food diaries overcome the within-person variation in food consumption, which is essential in women due to the significant weekday effect [60].

All participants were asked to register their typical consumption for seven consecutive weekdays and one weekend day. The seven days diet record was administered to study participants, with detailed instructions for filling in their diary.

For each day, the subject had to record six eating occasions (breakfast, morning snacks, lunch, afternoon snacks, dinner, and evening snacks.), a detailed description of the date, time of the meal, the menu, the food types and drinks (i.e., use of whole, semi-skimmed, or skimmed milk, the type of fish consumed, etc.) the food preparation methods, the ingredients of mixed dishes, recipes and the brand name of commercial products where appropriate. The portion sizes were expressed as common household measures (e.g., bowls, cups, plates and glasses), standard units or units such as grams or liters). A photograph aids for standard Moroccan household measures [57], and an example of one correctly filled in the day was also provided to participants to help them record precise quantities of food consumed. Participants were also requested to indicate if they took a vitamin D or multivitamin supplement, consistent with the FFQ or the EDR assessment.

For the estimate of vitamin D intake, the diet records were linked to the same food composition databases as for the FFQ (CIQUAl food composition database, the USDA) [54,58].

### 2.4. Sun Exposure Questionnaire (SEQ)

Dosimetry is the optimal method to assess sun exposure. However, a sun exposure questionnaire may provide similarly result, as statistically significant correlations have been reported between the two methods [61]. Thus, in our study, we developed the SEQ to objectively evaluate participants’ sun exposure during the last month and to address related environmental factors that account for the strength of UVB rays in the Moroccan context.

In summary, the SEQ (see in Supplementary Materials, Table S2) was designed according to previous studies [62,63,64,65,66,67]. It includes three domains of modifiable factors that influence vitamin D3 production in the skin. The indoor and outdoor sun exposure factors reflect time, frequency of sun exposure and body parts exposed to the sun. The tired domain describes participant’s different sun protection behaviors. All domains compute 15 items, and each item is scored on a scale of 0 to 4.

The sun exposure score (SES) is calculated as the product of the summed domains factors rating values, multiplied by one on the five points score attributed to each non-modifiable factor which are participant phototype (type I to type VI) and weather characteristics during the study period.

As a result, the SES was used to stratify participant sun exposure levels according to a score of 0 to 30. The sun exposure level was considered as insufficient if SES < 17, moderate if SES = 7.5 to 15, sufficient if SES = 15 to 30 and very sufficient or high if the score is > 30 [67].

To ensure the questionnaire’s clarity and comprehensibility, 30 university students who were not enrolled in the study took part in a pretest. Afterwards, the questions were revised based on the outcomes of the pretest.

The final questionnaire was administered to the sample population. The reliability of the questionnaire was assessed by internal consistency using Cronbach’s alpha, with an acceptable cutoff value of 0.7 [68].

### 2.5. Vitamin D Status Biomarker Assessment (25(OH)D)

The serum 25(OH)D analysis took place in a medical analysis laboratory of Mohamed V hospital in Meknes using electrochemiluminescence protein binding assay (ECLIA) using Roche Diagnostics, Cobas e411 analyzer. Registered nurses collected venous blood samples in EDTA tubes from all the participants in the study. All tubes were centrifuμged, and serum was stored at −80 °C until analysis.

A serum 25(OH)D concentration <20 ng/mL was considered a vitamin D deficiency, whereas a serum 25(OH)D concentration <30 ng/mL but >20 ng/mL define an insufficiency. Optimal status was considered a serum 25(OH)D ≥ 40 ng/mL [69] which is the level found in humans living naturally in sun-rich environments [70,71].

### 2.6. Anthropometry Measurement

Anthropometric measurements were performed while participants were minimally clothed and without shoes. The weight in kilograms was measured with a digital scale (SECA^®^), with a precision of 0.5 kg. The calibration of the devices was carried out using a known weight. Height was measured with a portable stadiometer (SECA 214) to the nearest 0.1 cm. Body Mass Index (BMI) was calculated as weight in kilograms divided by height in meters squared. Anthropometric status was categorized using classification according to BMI as follows: Underweight: BMI<18.5kg/m^2^, Normal: BMI: 18.5–24.9 kg/m^2^, Overweight: BMI 25–29.9 kg/m^2^, and obese ≥30 kg/m^2^ [72].

### 2.7. Statistical Analysis

Before analysis, the data were checked for normality of distribution using Kolmogorov-Smirnov and Shapiro-Wilk tests (*n* > 50), respectively. Data were non-normally distributed. Consequently, only non-parametric tests were used during the analysis. Descriptive data analysis was reported as medians, interquartile range in addition to means, standard deviations (SD) and percentage where applicable.

A range of statistical methods was conducted to assess the validity of the VitD-FFQ against the 7d-FR and the biomarker.

Firstly, Wilcoxon signed-rank test was used to compare the mean intake differences between the VitD-FFQ and 7d-FR. An effect size (r) was calculated where a large effect was 0.50 or higher, a medium between 0.30 and 0.5, and a small effect was between 0.10 and 0.30 [73].

The Bland–Altman scatterplots were used to investigate the level of agreement at an individual level visually. Bias and Limits of agreement (LOA) were calculated. We calculated the BA index based on how many differences lie outside the LOA. BA <5% was used as standard value for good agreement [74,75]. Regression analysis was also performed to quantify the proportional bias effect on the estimated intake difference between the VitD-FFQ and the 7d-FR [76].

Estimates from VitD-FFQ and each reference method (7d-FR and biomarker concentration) were grouped by quartiles for cross-classification (Individuals intakes belonging to the same quartiles, adjacent (±1) quartiles, or entirely misclassified (by ≥ two quartiles). It is recommended that at least 50% of participants are correctly classified, and less than 10% of participants are grossly misclassified into the opposite quartiles for each nutrient [77]. The weighted kappa statistic was used alongside cross-classification for another level of agreement. The weighted kappa statistic was calculated based on the observed and expected percentage of agreement from the cross-classification table. To interpret the kappa statistic the following standards were used: 0–0.20 = poor; 0.21–0.60 = acceptable; >0.61 = good [76,78].

Spearman Rank Correlation Coefficients were used to determine correlations between serum 25(OH) D concentrations, Vitamin D dietary intakes assessed by the FFQ and the 7d-FR, participants’ socio-demographic characteristics, SES and anthropometric measures (BMI). The magnitude of the correlation was set between −1 and +1, indicating the strength of the relationship. Correlation coefficients of (±0.8 to ±0.9 = very strong correlation, ±0.60 to ±0.70 = moderate correlation, ±0.20 to ±0.5 = fair correlation, and less than ±0.20 was a poor correlation[78].

Multiple linear regressions were applied to create models explaining 25(OH) D serum concentrations in all participants using potential predictor variables. The studied models were designed to compare, in particular the impact of vitamin D intake estimated by the VitD- FFQ and the 7dFR on the vitamin D status of participants. The linear correlation was studied between the dependent and explanatory variables using Spearman rank coefficient. Assumptions for the predictive models were tested by checking no multicollinearity (using variance inflation factors-VIF), multivariate normality and homoscedasticity of the residuals. A VIF > 10 was used to remove the variable from the model [77].

The triangular approach to validation, known as the method of triads, as described previously by Ocke and Kaaks in 1997 [29], was used to estimate validity coefficients between ‘unknown’ true nutrient intake and vitamin D intake estimated by the FFQ and 7d-EDR as subjective methods and the objective biomarker (25(OH)D concentration).

At first, the Spearman correlation coefficients were used to correlate vitamin D intake between the three dietary assessment methods (FFQ and EDR, FFQ and biomarkers, EDR and biomarkers). Next, the validity coefficient for the dietary assessment methods was calculated, and the estimated true ‘unknown’ vitamin D intake value (T) was according to the following equations:

ρ QT = √[(rQR ∗ rQB)/rRB]

ρ RT = √[(rQR ∗ rRB)/rQB]

ρ BT = √[(rQB ∗ rRB)/rQR].

where:

ρ = the validity coefficient, Q = FFQ; R = 7d-DR and
B = serum 25(OH) D concentration

The validation coefficient is considered as low (<0.2), moderate (between 0.2 and 0.6) or high (>0.6).

The calculated validity coefficient FFQ (ρQI) was considered as the upper limit and the correlation between the biomarker and vitD-FFQ was interpreted as the lower limit.

The 95% confidence intervals for the validity coefficients were estimated using bootstrap sampling, where 1000 samples of equal size (*n* = 152) were obtained with replacement from the study participants. In addition, we applied sensitivity analyses to adjust the approach of triads for covariates that were predictive of the serum 25(OH)D concentration among our participants [29].

All statistical analyses were performed in R version 3.4.2 (2017-09-28). A *p*-value of < 0.05 was considered significant for this study.

## 3. Results

### 3.1. The VitD- FFQ Items Presentation

The VitD-FFQ developed for this study included 78 items, as shown in Table 1.

Items of the VitD-FFQ consisted of 8 groups: dairy products and beverages, eggs, fish and sea products, meat and products, fat products (butter, margarine and oil), breakfast cereals, bakery and Moroccan biscuits, chocolate and cacao. In addition, two items were added at the end of the questionnaire to quantify vitamin D nutritional and other vitamins D-rich food consumption.

Example of the VitD- food frequency questionnaire for Moroccan women of reproductive age: items of cow milk and canned fish are provided as a supplementary file (see in Supplementary Materials, Table S1).

### 3.2. The Vit D-FFQ Validation

#### 3.2.1. Study Participant’s Characteristics

In total, 152 women of reproductive age were included in this validation study. They had good-quality FFQ and FR and gave a blood samples. Table 2 present the characteristics of the study participants. The median age was 25 years (IQR ±11; range 20–44). In this case, 14 women (9.22%) were employed at the time of the study, while 138 participants (90.78%) were mostly students (72.46%) and housewives (18.31%). The majority of the participants (73.68%) had a university education degree or higher, while 16.44% and 9.88% of them had, respectively, secondary level and primary one. Most of the women participants (61.84%) resided in urban while the remaining (38.15%) resided in rural areas.

The median length of our female participants was 1.64 m (IQR: ±0.06), while the median weight was 70 kg (IQR: ±17). Thus, the median BMI was 26.45 (IQR: ±7.67), with the majority of participants (75%) classified as having, respectively, overweight (45%), obesity (20%) and severe obesity (10%).

#### 3.2.2. Sun Exposure Scores and Related Factors

The SEQ (Table S2) achieve an acceptable internal consistency (Cronbach’s alpha = 0.73).

The median SES was 15.75 (±8.25) as shown in Table 3. The participant median (± IQR) scores for SEQ domains factors 1, 2, and 3 were 9.00 (±2.00), 16.50 (±10.75) and 6.00 (±5.00), respectively. Scores for each domains factor and the total SES increased significantly as sunlight exposure increased from low to high (all *p* < 0.001). When the SESs were interpreted, more the vast majority (96.37%, *n* = 147) were classified as having, respectively (55.26%, *n* = 84), sufficient and moderate (41.44%, *n* = 63) sunlight exposure (see in Supplementary Materials, Table S3).

#### 3.2.3. Vitamin D Status, Dietary Intake of Vitamin D Estimated by the VitD-FFQ and the 7d-FR

The median dietary intakes were 6.33 ± 5.02 and 7.10 ± 6.95 for 7d-FR and FFQ (Table 4). The vitamin D intakes determined from participants’ 7d-FR ranged between 3.01 and 25.96 µg/ day, whereas intakes estimated using the FFQ ranged from 3.28 to 21.60 µg/ day. None of the women participants in the study reported taking a vitamin D3 supplement.

In order to evaluate the change in vitamin D estimated intake between the two methods, a Wilcoxon sign rank test revealed a statistically insignificant difference in median vitamin D intake between the FFQ and the 7d-FR (Z =−1.538, *p* < 0.05). Thus, based on the positive Wilcoxon rank, the FFQ appears to overestimate the study participants’ vitamin D intake by 24 (15.78%). However, the overall effect size is small (r = 0.08) (Table 4).

Based on the average derived intake of the VitD-FFQ (8.77 (±4.98) μg/d) and 7d-FR (8.16 (±4.78) μg/d) within the validation study sample, respectively, 90.78% and 84.21% of participants had vitamin D intakes below the recommended daily allowance (RDA) (15 μg/day) by the Moroccan Ministry of health as well as the European Food Safety Authority. Moreover, 62.5% and 70.72% of participants’ reported intakes (in the FFQ and the 7d-FR, respectively) were below the world health organization and Institute of Medicine (IOM) recommendations (10 μg/d).

The major contributor to vitamin D intake in the VitD-FFQ was fish and seafood (52.90%), followed by milk and dairy products, including yoghurt and processed cheese (15.36%), eggs (11.36%), meat and meat product (8.45%) and fortified food (margarine, vegetable oil, and butter) (4.6%).

**Figure 2:**
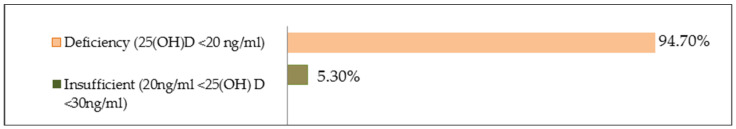
Vitamin D status in women participant in the study (*n* = 152).

Median (IQR) serum 25(OH)D concentrations for the study sample were 8.48 ± 6.55, and the average (SD) was 9.35 ((±5.38). The values ranged between 3–29.29 (ng/mL) (Table 4). Thus, all participants had a low level of serum circulating 25(OH) D, in which 144 (94.7%) participants were classed as deficient (<20 ng/mL). The remaining 8 (5.3%) participants were insufficient (20 ng/mL <vit D <30 ng/mL) (Figure 2).

#### 3.2.4. Association between Vitamin D Status, Dietary Intake, Sun Exposure Score, BMI and Socio-Demographic Characteristics of the Participants

Serum 25(OH) D status of all participants was significantly associated with vitamin D intake estimated by the FFQ (rho = 0.46), the 7d-EDR (rho = 0.36) and the total sun exposure score (rho = 0.36) (all *p* < 0.01). The strength of those correlations is fair (0.2< r < 0.5). Geographic localization and BMI were negatively associated with vitamin D status (respectively, rho = −0.15, rho = −0.17, *p* < 0.05). However, this correlation appears to be poor (rho < 0.20). No significant associations were shown between vitamin D status and socio-demographic participant’s characteristics (age, employment and education degree) (Table 5).

#### 3.2.5. Predictors of Serum 25(OH) D

Table 6 displays the association between participant serum 25(OH)D levels and explanatory variables as established by the regression analysis. The two models identified distinctively three variables as significant predictors of serum vitamin D concentration. VitD-FFQ vitamin D intake, sun exposure score, and urban localization are combined in the first model. In contrast, 7d-FR vitamin D intake, sun exposure score and BMI are combined in the second. Each model explains significantly 47% of the variance (*p* < 0.001) in serum vitamin D concentration. In addition, serum 25(OH) D concentration increased further when vitamin D daily intake was assessed by the VitD-FFQ rather than the vitamin D intake reported in the 7d-FR. 25(OH)D concentration increased by +0.56 ng/mL for each +1 uμg/day vitamin D intake (FFQ) versus +0.42 ng/mL for each 1 uμg daily vitamin D intake (7d-FR). Similarly, each positive unit change in the total sun exposure score may increase the 25(OH) D concentrations almost equally in the two models (+0.32 ng/mL in model I versus +0.34 ng/mL in the model II. The sun exposure score seems to be the most contributing variable in the vitamin D status over the two models (R = 0.47, *p* < 0.001). In model I, participants who live in an urban area may also have higher vitamin D levels +3.17 ng/mL. In model II, a 1 kg/m^−2^ increases in BMI may reduce serum 25(OH) D concentration of −0.16 ng/mL.

### 3.3. Criterion Related Validity of the VitD-FFQ

#### 3.3.1. Bland-Altman Analysis between VitD-FFQ and 7d-FR

**Figure 3:**
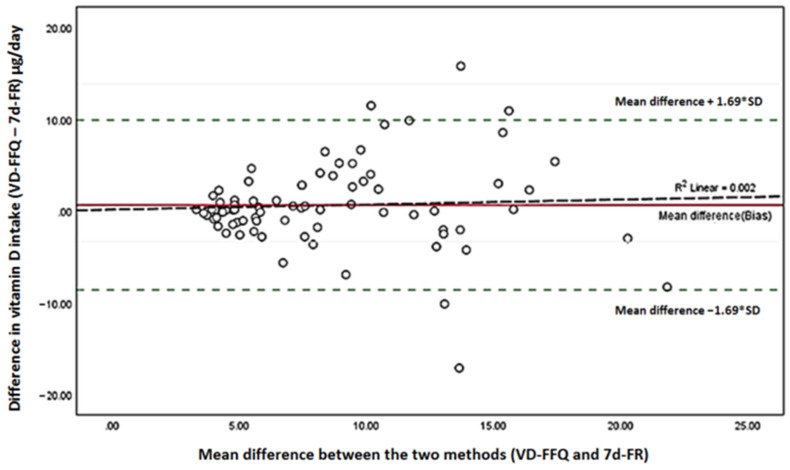
Bland–Altman plot showing agreement between food frequency questionnaire (FFQ) and seven days food record (7d-EDR) for vitamin D daily intake in 152 participants.

Figure 3 presents the Bland Altman agreement plot. Overall, there was an agreement between vitamin D intake from FFQ and vitamin D intake from 7d-FR. The bias between the vitamin D intakes from the two methods was 0.60µg/day. The upper agreement limit was 9.81, and the lower agreement limit was −8.61. Approximately 96.71% of vitamin D intakes were within the agreement limits. Only 5 (5/152) outlier observations (Four positives and one negative) occurred outside the 95% agreement range for the nutrient intake. Thus, the BA index is 3.29%. No correlation between the mean and the difference in vitamin D estimated intake using the 7d-FR and the VitD-FFQ was found in the linear regression analysis (R^2^ = 0.002, *p* = 0.545), indicating that there is no proportional bias (i.e., the mean vitamin D intake increases as the differences between the means increase).

#### 3.3.2. Cross-Classification and Weighted Kappa Cross-Classification of Vitamin D Intake into Quartiles by VitD-FFQ and Validation Methods (7d-FR and Status)

The quarterly categorization of vitamin D intakes and concentration distribution was used to assess the consistency between the three methods of estimate: VitD-FFQ, 7d-FR and 25(OH) D (Table 7). For participants in the same quartile, the serum concentration of vitamin D compared to the intake estimate from the VitD-FFQ recorded the lowest value (45.39%). The percentage of participants classified in the same quartiles according to the intake estimated by the VitD-FFQ and the 7d-FR was 59.21%. However, the cross-classification test reflects the agreement at the individual level. In contrast, the opposite quartile (%) showed that intake assessment by the VitD-FFQ and the two validation reference methods (7d-FR, the objective biomarker) had good validity at the individual level (≤10%).

The weighted kappa showed an acceptable classification agreement at the individual level, respectively, between both the VitD-FFQ and7d-FR (0.37, 95% CI: 0.27–0.46) and the FFQ-25(OH)D (0.28, 95% CI:0.18–0.38) (Table 6). A result between 0 and 1 for the weighted kappa coefficient is commonly expected agreement (excluding chance) at the individual level. Negative numbers imply an agreement “worse” than can be predicted by chance alone, whereas values of zero or near zero indicate “no more than pure chance” [78].

### 3.4. Application of the Method of Triads Model

The results from the method of triads are presented in Table 8. A significant correlation was clear between each of the three measurements in the method of triads, suμggesting a moderate association between estimates of vitamin D intake by VitD-FFQ and 7d-EDR (rQR = 0.64, 95% CI −0.53–0.73, *p* < 0.01) and a fair association between VitD-FFQ and participants 25(OH)D serum concentrations (r QB = 0.46, 95% CI −0.32–0.59, *p* < 0.01)) as between and vitamin D intake form the 7d-FR (rRB = 0,36, 95% CI −0.36–0.50, *p* < 0.01)) and 25(OH)D concentrations.

**Figure 4:**
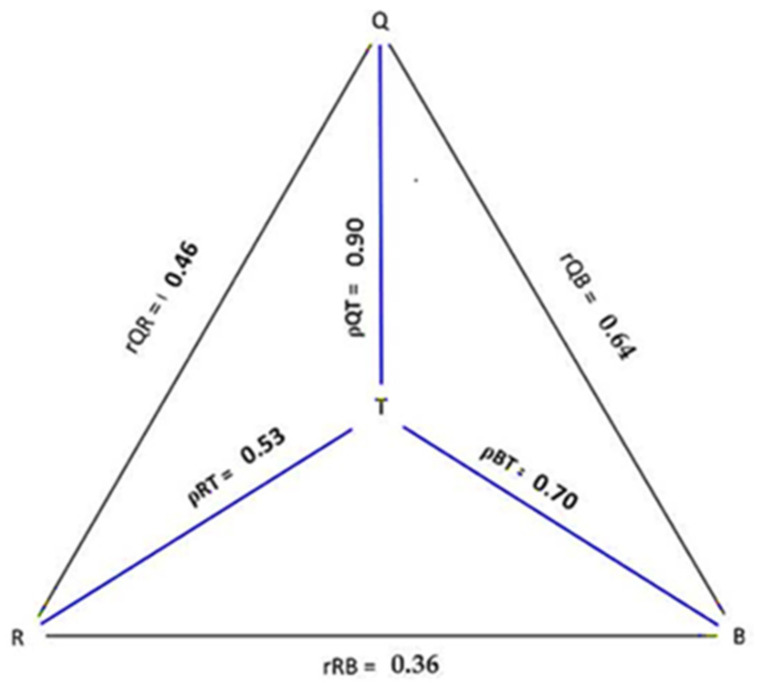
Graphical representation of the method of triads between vitamin D intakes estimated by the VitD-FFQ (Q), seven days Food record = R and Biomarkers = B of vitamin D status (serum concentration of 25-hydroxyvitamin D(25(OH)D)) for women participant (*n* = 152).

The overall validity coefficient calculation for the VitD-FFQ with true intake was 0.90 (95%CI: 0.89–0.92), indicating high validity. The validity coefficient between FFQ and the true intake range from 0.46 to 0.90. Whereas the validity coefficients for the 7-DR and biomarker -serum 25(OH)D were 0.70 (95%CI: 0.58–0.78) and 0.53 (95%CI: 0.35–0.63), respectively (Table 8 and Figure 4).

Additional adjustment for covariates (sun exposure score and BMI) of the participants improved the coefficients for the biomarker (validity coefficient 0.63 (95% CI 0.39–0.82) but had no considerable effect on the estimates for the other validity coefficient of the VitD-FFQ that remain high (0.72, 95% CI 0.51–0.92).

## 4. Discussion

Regarding the prevalence of hypovitaminosis D among Moroccan women and its possible implications on reproduction, assessing the dietary intake of this nutrient is critical for surveillance, mainly when undertaken with an adequate and well-validated FFQ.

In terms of usability, the newly developed FFQ is a semi-quantitative, self-administered and does not require specialized nutritional skills to be complete. Thus, we presume that this FFQ is practical for epidemiological research to reduce the cost of hiring interviewers and to elude social desirability bias in participants’ responses that interviewers’ presence may promote [28]. The ease of administration of this FFQ is enhanced by incorporating portion size based on standard Moroccan household measures and local market product packaging. Participants could complete this FFQ within 20 to 30 min which seems to be the average time appropriate for FFQ administration [28]. The VitD-FFQ food groups were organized on a list rather than meals, as neither option significantly affected nutrient estimates from the questionnaire in the literature [79]. The number of food items in the VitD-FFQ is within the range recommended for FFQ construction [28] and agrees with other FFQs developed to assess vitamin D intake in women [32,33,80,81,82] where items length ranged from 4 [80] to 161 food items [82].

As no accepted gold standard for measuring dietary intake exist, the errors of the two dietary assessment methods employed in the current study had to be as independent of one another as possible [27]. Food diaries allow for the long-term monitoring of participants’ eating patterns and account for any potential variations in dietary habits between weekdays and weekends [83]. Previous studies have utilized food diary methods, biomarkers of serum 25(OH)D concentration, or both to validate vitamin D-specific FFQs in women and various populations [30,31,32,33]. Thus, the validity of the VitD-FFQ was evaluated against criterion of 7d-FR and vitamin D status biomarker according to the method of triads.

Our results show a mean vitamin D intake assessed by FFQ of 8.77 (±4.98) uμg/day, similar to the estimated intake in other validation studies [30,32,84]. However, the median of the FFQ was 7.10 ± 6.95 uμg/day and seems to be higher than studies with published data for adult women from Qatar (5.98 uμg/day) [80], Libya (5.1 ± 5.7 uμg/day) [85], Korea (5.13 ± 3.50 uμg/day) [82] Ireland (4.91 uμg/day) [35], Uk (5.4 ± 7.7 uμg/day) [34], and Serbia (2.7 ± 1.7 uμg/day) [33]. This discrepancy may be attributed to differences in the participant’s age [28], which was broader in some studies [35,81], and the choosing time frame of diet recall [28] that reflected consumption over the 3previous months [85], six months [34,35] and 12 months in [82], compared with last month reporting intake in our study. Moreover, in other studies, we attempted to shorten the FFQ food items to only four main rich vitamin D foods [80], which may lead to underestimating nutrient intake [86].

A consistent finding among published studies is that vitamin D intake estimates by FFQs tend to average 3.12% [33] to 51.59% [34] higher than intake estimates by reference methods, including food diaries obtained over 3 to 14 days [32,34,35,80,81]. In line with this, our results showed an overestimation of FFQ-derived vitamin D compared with the FR estimates (+10.84%) that referred, however, to a small effect size (r = 0.08). A possible explanation for these differences is that respondents’ dietary vitamin D intake from 7days-FR was underestimated because they did not consume vitamin D-rich food during the research validation days.

In addition, the results from the Bland–Altman plot support a good agreement between the two methods of dietary intake (VitD-FFQ and 7d-FR). The BA index was 3.29%, which is <5%, a recommended standard for validation [74,75]. Similarly, in a validation study of vitamin D-specific FFQ against three days FR, conducted among Qatari women, Ganji et al. obtained a BA of 3.23% [80]. Park et al. compared two FFQs, the Korean calcium assessment tool (KCAT) and the Canadian calcium assessment tool (CAT), to estimate calcium and vitamin D intake in Korean women. The results showed a BA index of 3.1% for women under 50 and 3.9% in the total population, demonstrating an agreement between the FFQ and the reference method [82]. Moreover, the estimates of vitamin D intake obtained by the VitD-FFQ were comparable to those from the 7d-FR recalls (small mean difference) with no observed proportional bias [74]. The agreement interval approximately was near the national recommended daily vitamin D intake of 15 uμg/day. These findings indicate that the developed VitD-FFQ may perform consistently in populations with greater mean intakes and capture population intakes at or above the RDA threshold. Furthermore, this result makes our FFQ particularly appealing for application in epidemiological screening for dietary vitamin D intake inadequacy.

The dietary vitamin D intakes reported by either dietary assessment tool were generally low, and did not meet in the majority of the participant, the reference intakes recommended by international and national guidelines. Consequently, all women participants in this validation study present a hypovitaminosis D condition (25(OH)D < 30 ng/mL) and significant rate of vitamin D deficiency (94.7%) consistently with previous national studies [38,39,87]. The entire vitamin D participant intake, however, was obtained from natural food sources with no reported use of dietary supplements. These results align with other studies revealing that consuming vitamin D-rich food without food supplements is insufficient to cover physiological needs in most cases (97% to 100%) [82,83,84]. Indeed, according to 14 randomized control trials conducted in the MENA region and published between 2012–2017, supplementary doses of 1000–2000 IU/d may be necessary to reach a desirable 25(OH) D level at the target of 20 ng/mL [88].

On the other side, as stated earlier, the Moroccan food market provides affordable fortified food products to which vitamin D is added voluntarily, such as margarine breakfast cereals, processed cheese and yoghurt. However, it should be noted that no formal control is conducted on this product to determine its exact nutrient content. As a mandatory practice, 340 IU/liter of vitamin D3 is added to milk. This level corresponds to 17% of RDA [52]. In addition, 90% of edible oil is fortified with vitamin D3 at a rate of 300 IU/g, which satisfies 30% of daily needs [53]. However, the consumption of all fat-fortified products (margarine, vegetable oil, and butter) in our study sample represented only 4.6% of the total intake. In contrast, animal sources food (fish and seafood, milk and dairy product, eggs, meat and meat product) had a dominant contribution to the total daily vitamin D intake, assessed by the VitD-FFQ. Our participants’ young age profile (mean age: 26.6 ± 6.66 years) may support the findings, indicating a generational shift away from traditional Moroccan dietary models and toward a more westernized dietary pattern in which dairy products are increasingly contributing to intake [89]. Much more, several studies concluded that vitamin D was bioavailable in fortified milk and yoghurt and improved 25(OH) D status as well as markers of bone turnover [90,91]. Hence, the nutritional value and the resulting position of milk and milk products in the daily diet of youth highlight in our context the need for increased fortification of these products. The current fortification levels are likely insufficient to meet physiological requirements even in countries where vitamin D food fortification is mandatory [92].

The vitamin D status remains dependent on many factors [15] Above all else, we underlined individual sun exposure that greater affects the cutaneous production of 25(OH) D and may have a more significant impact on vitamin D status than dietary sources [14]. In the current study, we employed a specially designed questionnaire (SEQ) to assess participants’ UVB exposure while estimating their food intake over the previous month, which corresponded to the early spring season (March–April). The computed SES showed a fair significant correlation with 25-OHD levels (rho = 0.36, all *p* < 0.01) as our data indicated that the extreme majority of the participant was classified as having moderate (41.44%) to sufficient sunlight (55.26%) and the 25-OHD levels in the different categories of sunlight exposure (insufficient, moderate, sufficient, and high) significantly increased as sunlight exposure moved from moderate to high levels (*p* < 0.001) (see in Supplementary Materials, Table S4). Evenly, our finding is consistent with the previous research when a questionnaire scoring system was used. In a tropical setting, a study by Mansibang et al. (2020) on 75 adult Pilipino showed that most participants (80%) had moderate sunlight exposure in summer. A significant correlation was computed between the sun exposure score and 25(OH) D (r = 0.396, *p* < 0.001) [93]. Furthermore, the average serum 25-OHD was higher in each of the sunlight exposure groups as compared to our findings (low sunlight exposure group 17.51 ± 5.96 ng/mL; moderate sunlight exposure group 26.78 ± 6.55 ng/mL; high sunlight exposure group 30.97 ± 5.88 ng/mL) [93]. Among a South Asian population and subtropical sitting in Pakistan, Humayun et al. found that the mean short-term sun exposure questionnaire scores had a fair correlation to the 25-OHD levels in summer (r = 0.36) and winter (r = 0.43) [94]. Such comparisons may lead to misinterpretation, due to differences in locations and seasons of the conducted studies [95]. In addition, measuring sunlight exposure is complex, as every personal and environmental factor increases the chance of inaccuracy [96]. The association between individual UV exposure and serum 25(OH) D concentrations when using a questionnaire is expected to be low [96].

Furthermore, we developed two predictive models for vitamin D concentration, comparing the contribution of vitamin D intake of the VitD-FFQ and the 7d-FR in the 25(OH)D concentration variance. We account, in addition, for all participants’ characteristics and factors that may influence serum vitamin D concentration. The set of predictors included in our final models explained about 47% of the total variability in vitamin D concentration. The most consistent contributor to the predictive ability of the vitamin D status models was sun exposure. Evenly, all the predictors (BMI, urban localization and sun exposure) found herein had various association strengths with the vitamin D concentration and have been identified as consistent predictors of low 25(OH) D levels across the lifecycle in the MENA region [88]. In particular, we demonstrated that natural food vitamin D intakes were, significant predictors of vitamin D status and that the FFQ performed better than the FR in the objective measures’ prediction. The FFQ intake account for 56% of 25(OH) D levels variance while 7d-FR explain 36% of the variance. Such finding was not reported previously. In the one published validation study that performed a regression model to explain vitamin D status among athletes, total dietary vitamin D intakes assessed by a three-month recall FFQ and seven-day food records, as well as vitamin D supplement intakes, were unable to predict vitamin D status across all time seasons. BMI and tanning bed were the only predictors in winter and spring [84]. Main raisons that must be considered to interpret theses different results are associated to the food products fortification amount adopted in each country and variation in eating habits. Thus, using serum 25(OH) D concentrations as a biomarker for FFQ validation is challenging and has its limitations due to the influence of several factors other than diet.

In FFQs validation studies, correlation coefficients greater than 0.7 are uncommon. This phenomenon is known as the “ceiling of validity” and is attributed to the fact that a structured questionnaire cannot fully capture the inherent complexity of the human diet [97]. According to this, studies showed that for most nutrients, correlation coefficients with biomarkers are 0.3 to 0.5 [28]. Similarly, in our result, the absolute Spearman rank-correlation coefficient between total vitamin D intake assessed by the FFQ and biomarker showed a significant moderate association (r = 0.46). However, correlations were slightly better between FFQ and biomarker than between EDR and biomarker (rho = 0.36), which is reasonable because the FFQ and EDR are two methods that estimate dietary intake while, the biomarker status can be influenced by more factors than diet, such as sun exposure. Herein, it is essential to consider the different timeframes for each dietary exposure measure in this study. The FFQ expresses dietary habits over the past month, the EDR on the intake of one week, and the serum biomarkers are likely to represent the preceding month of dietary intake considering the half time of 25(OH) D in 15 days. Thus, the true EDR correlations may be underestimated due to differing time frames. Moreover, there may be another factor, such as the high educational level of our research participants (university degree in 73.68 % of cases), which generally affects recall of diet by FFQs [28].

In addition, vitamin D intake estimated by the FFQ showed moderate (rho = 0.63) levels of relative validity compared with the 7d-FR. This correlation was consistent with the strength of correlations observed in previous validation research (using food records), reporting a correlation coefficient of 0.56 [34,98]. Far more, cross-classification analysis, in contrast to correlation, could provide accurate and unbiased sight of how the FFQ performed [27]. In our results, the cross-quartile data agreement at the individual participant level between the intake assessment by the VitD-FFQ and the 7d-FR and serum 25-(OH)D biomarkers was 10% gross misclassification (opposite quartile), with computed kappa values (k) of 0.37 and 0.28, respectively, indicating acceptable classification agreement. These results support the ability of the FFQ to adequately rank women participants to their vitamin D intake categories in a similar way as the reference methods [76] and exclude pure chance in the interpretation of the predicted agreement [78]. Moreover, our findings are consistent with prior validation studies that included female participants and used a food record [32,82] or both 24h recall and biomarker as reference methods [85].

The validity coefficients between the ‘true’ intake (I) of vitamin D and for the FFQ (ρ QT = 0.90) as well as the 7d-FR (ρ RT = 0.70) were higher than those for the serum biomarker (ρ BT = 0.53) which was moderate. Moreover, the observed validity coefficient for the FFQ tended to be higher than those for both the dietary reference method (FR in current research) and the biomarker. Our results are similar to most studies that used the method of triads model for validation of vitamin D FFQ among women of reproductive age: ρ QT = 0.84 [33], and as well as among mixed groups of man and women ρ QT = 0.92 [34], ρ QT = 0.88 [35]. The method of triads model assume that random errors between the three methods are independent and that all three measurements have a linear relationship with the unknown true intake [29]. Violation of this assumption is more common when 24hR or food records are used as reference methods since they all, as the FFQ, rely on self-report. Positively correlated measurement errors may lead to an overestimation of the validity of the FFQ [29]. Using data from self-reported questionnaires and nutritional biomarkers should provide extensive insight when quantifying dietary vitamin D FFQs and FR. According to Kaaks et al. [30], althouμgh biomarkers can be measured more objectively, they do not necessarily reflect dietary intake better than self-reported intake. Only a few recovery biomarkers (i.e., 24 h urinary nitrogen for protein intake) directly reflect quantitative dietary intake. However, most nutritional biomarkers, such as vitamin D, which are concentration markers, are presumed to be associated with intake but do not reflect absolute nutrient intakes. Moreover, differences in biomarker concentrations can occur due to intrinsic variability (i.e., bioavailability and metabolism) [28], genetic [99] and environmental factors [96].

Based on this fact, we considered the validity coefficient FFQ (ρQI) as the upper limit and the correlation coefficient of FFQ and biological markers (rQB) as the lower limit of the validity coefficient between FFQ and the true intake. With this approach, we observed a lower limit of the validity coefficient of 0.46 and an upper limit of 0.90. Hence, the overall validity varies from moderate to high and further demonstrates that our developed FFQ is valid for assessing vitamin D intake in Moroccan women. Furthermore, the 95% confidence intervals for the validity coefficients in our study are narrow, and the upper limit for all intervals is lower than 1. This strength reflects the accuracy of the validity coefficient and an absence of random sampling variations between dietary methods or violations in the underlying assumptions of the model [27,43].

Moreover, after controlling for the most influencing confounding (Sun exposure and BMI), the strength of the biomarker’s validity coefficient improved from moderate to high (0.53 to 0.63). However, the validity coefficients of both dietary assessment methods (FFQ and FR) almost remain high (0.72) when controlling for participants BMI. However, to improve the intake estimation by the dietary methods, it is also imperative to adjust nutrient intakes for the total energy intake. Studies showed that subjects tend to underreport energy intake in FR and over-report energy intake in FFQ. Unfortunately, our FFQ was not designed for energy assessment, so we cannot account for potential dietary misreporting in the present study [100]. In other validation studies, we found a comparable association between total vitamin D consumption measured by the FFQ and the vitamin D biomarker using the energy-adjusted Spearman rank-correlation coefficient and the crud data [33].

We acknowledge that our study has some limitations. The primary limitations lie in the study’s cross-sectional nature due to time and logistic constraints. Thus, all data were collected on only one occasion for each participant. The FFQ was not validated in all four seasons, which may be essential in the study design, given the seasonal variation in vitamin D intake. In addition, we recruited a large sample of 150 women participant for a validation study using biomarker as reference method [44], but it may not represent Moroccan women regarding specific social characteristics such as education level. Indeed, illiteracy is common in Morocco’s general population, whereas most of our study sample was composed of students with a high level of education. Furthermore, a selection bias might have been present as a participant were all volunteers, mainly from the university community and were promised a vitamin D status result and medical advice at the end of the study. However, forcing unmotivated subjects to participate in the study may impact data quality [100].

The study had several strengths. First, the development and validation of vitamin D-specific FFQ was the first initiative in Morocco. Our results describe vitamin D dietary intake in Moroccan women, which correlates with vitamin D status for the first time. This information could be valuable in developing dietary guidelines, especially when no other types of relevant data are available. Another essential feature of the developed FFQ is that easy to use and practical. It includes a comprehensive list of fortified foods we attempted to collect in the local market to ensure representativeness and precision in estimating intakes based on product labels. Second, participants were trained and motivated by qualified professionals to complete the FFQ and the FR without contributing to misclassification, which is an essential factor that favorably impacts data collection quality [101]. The FFQ has also been developed in both French and Arabic, reducing the impact of language barriers and promoting self-administration.

Lastly, the major strength of our study is the multiple statistical approaches to assess agreement between methods and comparisons with previous studies using the method of triads that have demonstrated the completeness and validity of our FFQ. We assessed the agreement between VitD-FFQ and FR and the biomarkers with different statistical tests (i.e., BA plot, cross-quartile classification, weighted K, and correlation). Based on these tests, we achieved an acceptable to good agreement between the vitamin D intakes from VitD-FFQ and 7d-FR. More importantly, we validated the newly established FFQ using a triangular approach to overcome the dependent errors that may be present in dietary assessment methods (herein, the FFQ and FR methods). As a result, we reported on how individual risk factors affected the computation of the triad method’s validity coefficients and the predictive ability of 25(OH) D3 in regression models. We demonstrated a reasonable accuracy of estimates and interpretation of the results. Furthermore, the correlation between the circulating 25(OH) D concentrations and dietary intake assessed by the VitD-FFQ confirms that the significant confounding effect of sun exposure was effectively controlled, such that vitamin D status in our participants was attributed to food consumption. Further Moroccan research may benefit from using the established VitD-FFQ and the SEQ to generate detailed data on how sun exposure factors and vitamin D intakes may impact vitamin D status in women, particularly during the summer season.

## 5. Conclusions

In this validation study applying the method of triads, results indicate that the developed VitD-FFQ demonstrated high criterion validity for estimating absolute vitamin D intake among Moroccan women of reproductive age. This FFQ can serve as a valuable tool in research to assess usual dietary vitamin D intake and identify women at risk for vitamin D deficiency, particularly in resource-limited settings. Further studies, however, are required to determine the FFQ reproducibility and its validity in other groups of the Moroccan population. The study showed, in addition, a high prevalence of vitamin D deficiency in the study sample, strengthening the epidemiological evidence that vitamin D deficiency is widespread in Moroccan women and dietary intake is an essential determinant. A national strategy is needed to address this public health concern.

## Figures and Tables

**Table 1:** Number of items selected for the VitD-FFQ.

Food Groups	Items	Number of Items
**1-Dairy Products and beverages**	**Cow milk:** Vit D3 fortified (pasteurized, whole fat, 3.5% fat) Ultra high temperature whole cow milk vit D fortifed Semi skimmed milk (Milk, cow, partly skimmed, 1.5% fat) Whole fat powdered Milk , reconstituted with water Farm-fresh cow’s milk Yogurt (whole fat, nature). Light yogurt (0% fat) enriched with vitamin D3 Yogurt flavored or with fruit, sweetened, non-reduced fat Yoghurt flavoured, sweetened, with cream Milk drink, or drinking yogurt, flavored or with fruit, sweetened. **Soy drinks**: vitamin D3 and calcium fortified **Processed cheese:** Hard cheese (Emmental, Cheddar, Comte, Abondance) Hard cheese (Gruyere, Masdam) Hard cheese (Parmeson, Requefort) Semi-hard cheese (Gouda, Edam, Munster) Soft cheese (Camembert, Brie) feta- cheese (in oil or aromatic) Cottage cheese natural or aromatic Fresh cheese (ricotta, Mozarelle,) Melted cheese in portions or spreadable cubes	21
**2-Eggs**	Pan-fried egg with added fat Pan-fried egg without added fat Crambled eggs Boiled egg Poached egg.	5
**3-Fish and Sea Products**	Fatty fish fresh and frozen (Sardin, truit, salamon, hareng-ranga, mackerel-cabaila, swordfish-chbada, halibut, chinchar–chren, eel –salbah, carp, perch, tuna…) White fish fresh and frozen (sole, whiting, sea bream…) Code liver Canned fish, smoked fish, and salted fish Shellfish (calmer, scampi, shrimps, mussels)	15
**4-Meats Products**	Red meat (Beef, veal, sheep..),eaten with or without fat Poultry (chicken and turkey) Organ meats (liver, kidney) Sausage or merguez Smoked meats or charcuterie (chorizo, mortadella, salami, ham, casheer, etc.)	15
**5-Fat Products**	Margarine vit D3 fortified Normal margarine (more than 70% fat) light margarine (40 to 60% fat) Butter Sweet butter pasteurized at 80% or 82% fat Light pasteurized unsalted butter (40 to 60% fat) Unsalted butter or low-fat fortified salt Semn **Vegetable oil vitamin D3 fortified:** Mixed table oil with 3 seeds: sunflower, soya, colza Soya oil	10
**6-Breakfast cereals**	Cereal vit D fortified Other non-fortified Muesli (normal and crispy) and oatmeal vit D fortified.	3
**7-Backery and Moroccan biscuit**	Ordinary homemade cake or prepackaged Madeleine Viennoiserie (krachel, chocolate bread, raisin bread, croissant, brioche) Moroccan biscuits (thee dry biscuit, Fekkas, date biscuit)	3
**8-Chocolate and cacao**	Milk chocolate, bar Dark chocolate with 70% cocoa minimum, extra, tasting, bar Dark chocolate with dried fruits (hazelnuts, almonds, raisins, praline), bar Cocoa unsweetened, soluble powder for drinks Sweet cocoa or chocolate powder for drinks, enriched with vitamins and minerals	4
**Other vitamin D3 fortified product if applicable**	Type, quantity and frequency	1
**Food supplements(in vitamin D) used if applicable**	Type, dosage and frequency	1
TOTAL		78

**Table 2:** Descriptive characteristics of study participants (*n* = 152).

	Unit/Category	Study Participants (*n* = 152)
**Age (Years)**	**Median (IQR) Range**	**25.00 (±11.00)** **20.00–44.00**
**Employment:** **Unemployed:** Students Housewives **Employed:** Professor Liberal profession	%	90.78 72.46 18.31 9.22 7.90 1.32
**Education degree** Primary secondary University or higher	%	9.88 16.44 73.68
**Localization** Urban Rural	%	61.84 38.15
**Length (m)**	Median (IQR)	1.64 (±0.04)
**weight (kg)**	Median (IQR)	70.00 (±17.00)
**BMI (kg/m^2^)**	Median (IQR)	26.45 (±7.67)
**BMI classification by kg/m_2_** Underweight (<18.5) Normal weight (18.5–24.99) Overweight (25–29.99) Obese (≥30) Sever obesity (30–34.9)	%	1.30 23.80 45.00 20.00 10.00

**IQR**: interquartile range; **BMI**: body Mass index.

**Table 3:** Sun exposure score and sunlight categories assessed by the SEQ in 152 participants.

SEQ Domains Factors	Exposure Estimated Scores (*n* = 152)		Exposure Categories (Insufficient, Moderat, Sufficient, High) ^b^
	Mean ± SD	Median ± IQR	*p* Value ^c^
1.Indoor sun exposure	7.60 (±4.10)	9.00 (±2.00)	<0.001
2.Outdoor sun exposure	16.28 (±5.39)	16.50 (±10.75)	<0.001
3.Sun protection practices	6.71 (±2.64)	6.00 (±5.00)	<0.001
Sun Exposure Score (SES) ^a^	16.07 (±5.62)	15.75 (±8.25)	<0.001

^a^ Sun exposure score = overall domains scores × individual phototype score ×Average weather index during the reported duration of exposure (0.75). ^b^ Interpretation of exposure score in categories is as follow: insufficient: <7.5, moderate: 7.5–15 and sufficient: 15 to 30, high sun exposure (>30). ^c^ Kruskal Wallis H test. SEQ: sun exposure questionnaire; IQR: interquartile range.

**Table 4:** Vitamin D daily intake assessed by VitD-FFQ and 7d-FR 7 and 25(OH)D Serum concentration in 152 study participants.

	Mean ± SD	Rang	Median ± IQR	MD ^a^	Diffrence % (IQR) ^b^	*p* Value *
**Vitamin D intake(µg/d)**	8.77 (±4.98)	3.28–21.60	7.10 ± 6.95	0.77	10.84 (−0.27–4.36)	0.12
VitD-FFQ						
7d-FR	8.16 (±4.78)	3.01–25.96	6.33 ± 5.02			
25(OH)D (ng/mL)	9.53 (±5.38)	3–29.29	8.48 ± 6.55			

^a^ Median difference = median food frequency questionnaire–median food record. ^b^ Percentage of median difference was computed using the formula: (median FFQ median 7d-FR)/median FFQ × 100). * Wilcoxon signed rank test were used to compare values obtained from both FFQ and 7d-FR FFQ: food frequency questionnaire. 7d-FR: 7day food record, MD: median difference, IQR: inter-quartile range, SD: standard deviation VitD-FFQ: vitamin D food frequency questionnaire; 7d-FR: 7day food record; MD: median difference; IQR: inter-quartile range; SD: standard deviation.

**Table 5:** Association between Vitamin D Status, dietary intake, UV Exposure score, BMI and socio-demographic characteristics of the participants.

	VitD-FFQ	7d-FR	Sun Exposure Score	Age	Employment	Education Degree	Localization	BMI
**25(OH) D (ng/mL)**	0.463 **	0.36 **	0.36 **	0.13	−0.21	0.036	−0.15 *	−0.17 *

Spearman Rank Coefficients. ** Correlation is significant at the 0.01 level. * Correlation is significant at the 0.05 level.

**Table 6:** Significant predictors of Serum 25(OH)D concentrations in study participants.

	Predicators Variables	Coefficient B (SE)	(97.5% CI)		*p* Value
			Lower Limite	Upper Limite	
**Model I** **(R = 0.47,** ***p* < 0.001)**	**Vitamin D intake (VitD-FFQ)**	**0.56 (0.08)**	0.39	0.73	*p* < 0.001
	SES	0.32 (0.04)	0.23	0.41	*p* < 0.001
	Localization	3.17(0.86)	1.45	4.89	*p* < 0.001
**Model II** (**R = 0.47,** ***p* < 0.001)**	Vitamin D intake (7d-FR)	0.36	0.27	0.55	*p* < 0.001
	SES	0.47	0.25	0.43	*p* < 0.001
	BMI	−0.16	−0.31	−0.04	*p* < 0.001

B: regression coefficient; SE: standard error; CI: confidence interval; FFQ: food frequency questionnaire; 7d-FR: 7day food record, SES: Adjusted sun exposure score; BMI: Body mass index. Model I: 25(OH)D status = Dependent variable (Y). VitD-FFQ(X1), Total sun exposure score (X2) and geographic localization (X3) = predicators variables. Regression equation for predictive model 1: Y = 0.56X1 + 0.32X2 + 3.17 X3 − 8.58. Model II: 25(OH)D status = Dependent variable (Y). 7d-FR(X1), Total sun exposure score (X2), body mass index (X3) = predicators variables. Regression equation for predictive model II: Y = 0.42X1 + 0.34X2 − 0.18X3 − 0.78

**Table 7:** Cross-classification of vitamin D intake into quartiles by VitD-FFQ and validation methods (7d-FR and status).

Vitamin D Intake/Status Assessed by	Vitamin D Intake Assessed by VitD-FFQ			Weighted Kappa ^b^ (95 % Lower, Upper CI)
	Classified into Same Quartile (%)	Classified into Same ± 1 Quartile (%)	Classified into Opposite Quartile ^a^ (%)	
7d-FR	59.21	35.52	1.31	0.37 (0.27–0.46)
25(OH)D	45.39	34.21	3.94	0.28 (0.18–0.38)

^a^ Interpretation criteria (% in opposite quartile): good: ≤10%, poor: >10%. ^b^ Interpretation criteria: good: ≥0.61; acceptable: 0.20–0.60; poor: <0.20.

**Table 8:** Correlation coefficients between each of the three assessment methods and the validity coefficient calculated with the method of triads, Simple and adjusted for covariates ^a^.

	Serum 25(OH)D Concentration (*n* = 152)	
	Simple Correlation	Adjusted for Covariate ^a^
Correlation coefficients		
rQR (95%CI)	0.64 ** (0.53–0.73)	0.53 (0.38–0.68)
rQB (95%CI)	0.46 ** (0.32–0.59)	0.46 (0.31–0.59)
rBR (95%CI)	0.36 ** (0.21–0.50)	0.46 (0.29–0.60)
Validity coefficients		
ρQT (95%CI) range	0.90 (0.89–0.92) (0.64–0.90)	0.72 (0.63–0.81)
ρRT (95%CI) range	0.70 (0.58–0.78) (0.46–0.70)	0.72 (0.59–0.83)
ρBT (95%CI) range	0.53 (0.35–0.63) (0.36–0.53)	0.63 (0.48 0.72)

rQR: correlation between VitD-FFQ and 7d-FR; rQB: correlation between serum 25(OH)D biomarker and VitD- FFQ; rBR: correlation between 7d-FR and serum 25(OH)D biomarker; ρQT, validity coefficient of the VitD-FFQ; ρRT, validity coefficient of the 7d-FR; ρBT, validity coefficient of serum 25(OH)D biomarker; CI, 95% confidence interval. ^a^ All correlations and coefficients are adjusted for sun exposure score and BMI, Except for the association between VitD-FFQ and 7d-FR that was only adjust for the BMI. Range: The lower limit is rQR for the FFQ, rQB for the biomarker and rBR for the 7d-FR; the upper limit is calculated by the method of triads. ** Correlation is significant at the 0.01 level.

## Data Availability

The datasets generated and analyzed for the current study are available from Prof Filali-Zegzouti Younes, upon request.

## Supplementary Materials

The following supporting information can be downloaded at: https://www.mdpi.com/article/10.3390/nu15040796/s1, Table S1: Vitamin D-Food frequency questionnaire for Moroccan women of reproductive age; Table S2: Sun exposure questionnaire to assess sunlight exposure score in Moroccan women of reproductive age; Table S3: Sun exposure factors and total sun exposure score assessed by the SEQ in 152 participants; Table S4: Comparison of Median serum 25-OHD according to sunlight exposure score levels.
