# Supplementary material for: Development and Validation of Vitamin D- Food Frequency Questionnaire for Moroccan Women of Reproductive Age: Use of the Sun Exposure Score and the Method of Triad’s Model
Source: Nutrients. 2023 Feb 4;15(4):796. doi: 10.3390/nu15040796 (PMC9967684; doi:10.3390/nu15040796)
Supplement: Supplementary file 1 [file nutrients-15-00796-s001.zip › Table S1.pdf]

Vitamin D-Food frequency questionnaire for Moroccan women of reproductive age

Name:
Date:

In this food-frequency questionnaire, we ask about your eating habits over the last month to estimate your daily vitamin D consumption. Various food products (food groups) are listed in the table below. Please describe (as precisely as possible) how frequently you consume the mentioned products and indicate your average daily portion. Consider the meals consumed away from home.

For each item, please fill a box (with a cross) that best describe how often you consumed those items during the past month, then fill in the square near the choice that best describes your usual portion size.

Table S1. Example of the vit D- food frequency questionnaire for Moroccan women of reproductive age (items of cow milk and canned fish).

**Food groups**	**How often do you consume the following product?**						**Example portion sizes**	**What is the average portion per day?**
	**Never or less than once per month**	**1-3 times per month**	**1 time per week**	**2-4 days per week**	**5-6 days per week**	**Every day**		
**Cow milk:**<br>Vit D3 fortified (pasteurized, whole fat,3,5%fat: Central, Chergui,jibal,Jaouda)							One glass (type thea glass : ANBA) = 125ml<br>One big glass =225ml	☐ 125ml or less<br>☐ 125-250ml<br>☐ 250-375ml<br>☐ 375ml or more
**Canned fish :**<br>Sardin (in vegetal oil)<br>Sardin (in tomato sauce							2 canned sardines=40g<br>1box=120g	☐ 50 g or less<br>☐ 50 - 100 g<br>☐ 100 - 150 g<br>☐ 150 g or more
