# Supplementary material for: Development and Validation of Vitamin D- Food Frequency Questionnaire for Moroccan Women of Reproductive Age: Use of the Sun Exposure Score and the Method of Triad’s Model
Source: Nutrients. 2023 Feb 4;15(4):796. doi: 10.3390/nu15040796 (PMC9967684; doi:10.3390/nu15040796)
Supplement: Supplementary file 1 [file nutrients-15-00796-s001.zip › Table S2.pdf]

Table S2. Sun exposure questionnaire to assess sunlight exposure score in Moroccan women of reproductive age

Sun exposure score						
**Sun exposure domains:**						
	**Items**	**0**	**1**	**2**	**3**	**4**
**Indoor sun exposur**	Frequency of indoor sun exposure (in terrace, balcony, courtyard)	Never	1 time /week	2 to 3 times/week	4 to 5 times /week	More than 5 times /week
	Indoor exposed body part		Face	Face, hand	Face, arm	Face, arm, leg
	Indoor duration sun exposure	Less than 5min	5 to 15 min	15 to 30min	30 to 60min	More than 60 min
	Indoor sun exposure time slot	Before 7a.m.	7 a.m. to 9 a.m.	9 a.m. to 11 a.m. / 5pm to 19 p.m.	11 a.m. to 1 p.m.	1 pm to 5 pm
**outdoor sun exposur**	Frequency of professional or routine outdoor activities,	Never	1 time /week	2 to 3 times/week	4 to 5 times /week	More than 5 times /week
	A time slot for professional or routine outdoor activities	Before 7a.m.	7 a.m. to 9 a.m.	9 a.m. to 11 a.m. / 5pm to 19 p.m.	11 a.m. to 1 p.m.	1pm to 5pm
	Duration of professional or routine outdoor activities	Less than 5min	5 to 15 min	15 to 30min	30 to 60min	More than 60 min
	Outdoor exposed body part		Face	Face, hand	Face, arm	Face, arm, leg
	Practice outdoor activities (sports, hiking, walking, camping, swimming, et. c,)	Never	1 time /week	2 to 3 times/week	4 to 5 times /week	More than 5 times /week
	Duration outdoor activities	Less than 5min	5 to 15 min	15 to 30min	30 to 60min	More than 60 min
	Time slot for sun exposure in the open air	Before 7a.m.	7 a.m. to 9 a.m.	9 a.m. to 11 a.m. / 5pm to 19 p.m.	11 a.m. to 1 p.m.	1pm to 5pm
	Part of the body exposed in the open air		Face	Face, hand	Face, arm	Face, arm, leg

**Sun protection practices**	staying out of the sun	used shade of tree/building/ parasol etc.	Market in the shade	Car/bus/van smoked window/of	Car/bus/van Glass windows up	No protection
	Type of sunscreen used	SPF sunscreen of 50 and +	SPF sunscreen from 30 to 50	Sunscreen SPF from 15 to 30	Other creams and lotions without knowledge of the FPS or SPF 6 to 10	No sunscreen
	Type of clothing usually used	Usual clothes with long sleeves/long pants/heavy/ tight clothes with dark colorants	Usual clothes with long sleeves/long pants/ loose with dark colorants	Long loose clothing with light coloring.	Short sleeve/pants/shorts	Short no shirt /bikini
**Ajustement variables**						
		**0**	**0.25**	**0,5**	**0,75**	**1**
**Phototype**			Type V & VI	Type III &IV	Type II	Type I
**Weather outside**		rainy	cloudy/light rainy	cloudy	sunny/light cloudy	sunny

Sun exposure score = the sum of the domain scores x phototype score x outdoor weather score.

Interpretation:
The score is considered insufficient if the score is below 7.5, moderate if the score is between] 7.5 -15[, sufficient if the score is between [15 -30], and as very sufficient or high if the score is above 30.
