# Supplementary material for: Development and Validation of Vitamin D- Food Frequency Questionnaire for Moroccan Women of Reproductive Age: Use of the Sun Exposure Score and the Method of Triad’s Model
Source: Nutrients. 2023 Feb 4;15(4):796. doi: 10.3390/nu15040796 (PMC9967684; doi:10.3390/nu15040796)
Supplement: Supplementary file 1 [file nutrients-15-00796-s001.zip › Table S3.pdf]

Table S3. Sun exposure factors and total sun exposure score assessed by the SEQ in 152 participants

	Sun exposure domains	Total participants (n=152)		
		Frequency (n)	Relative frequency (%)	Median score (IQR)
Modifiable factors	**I-Indoor sun exposure factors**			
	**1-Frequency of exposure per week**	39	25.65	
	Never	31	20.39	
	One time/week	82	53.94	
	2 to 3 times/week			
	**2-Exposed body part :**	58	56.86	
	Face and hand	34	33.33	
	Face and arm	21	20.58	
	Face, arm, leg			
	**3-Duration of exposure :**			**9.00 (±2.00)**
	less than 5min/d	5	4.42	
	15 to 30min/d	12	10.61	
	30 to 60min/d	62	54.86	
	More than 1h/d	34	30.08	
	**4-Exhibition time slot**	5	4.42	
	6 a.m. to 8 a.m.	12	10.61	
	8 a.m. to 10 a.m.	62	54.86	
	10 a.m. to 12 p.m.	34	30.08	
	12pm to 4pm			
	**II-Outdoor activities**			
	**1-Practice of professional or routine outdoor activities**			
	**Frequency / week**	25	16.44	
	2 to 3 times/week	59	38.81	
	4 to 5 times /week	68	44.73	
	More than 5 times /week			
	**2-Exposed body part :**			
	Face and hand	140	92.10	**16.50(±10.75)**
	Face and arm	10	6.57	
	Face, arm, leg	2	1.31	
	**3-Duration of exposure :**			
	15 to 30min/d	16	10.52	
	30 to 60min/d	37	24.34	
	More than 1h/d	99	65.13	
	**4-Exhibition time slot**	4		
	6 a.m. to 8 a.m.	50	2.63	
	8 a.m. to 10 a.m.	75	32.89	
	10 a.m. to 12 p.m.	23	49.34	
	12pm to 4pm		15.13	

**Practice of outdoor activities sport / hiking etc..**			
**Frequency / week**			
Never	4	12.5	
One time/week	14	43.75	
2 to 3 times/week	12	37.5	
4 to 5 times /week	2	6.25	
More than 5 times /week			
**-Exposed body part :**	2	6.25	
Face and hand	22	68.75	
Face and arm	8	25	
Face, arm, leg			
**Duration of exposure :**	6	18.75	
15 to 30min/d	7	21.87	
30 to 60min/d	19	59.37	
More than 1h/d			
**Exhibition time slot**	14	43.75	
6 a.m. to 8 a.m.	16	50	
8 a.m. to 10 a.m.	0	0	
10 a.m. to 12 p.m.	2	6.25	
12pm to 4pm			
**Sun protection practices :**			
**Staying out of the sun**			
Market in the shade	36	23.68	
Car/bus/van smoked window/of No	59	38.81	
protection	56	36.84	
**Sunscreen use**			**6.00 (±5.00)**
SPF sunscreen from 30 to 50	36	23.68	
Sunscreen SPF from 15 to 30	56	36.84	
No sunscreen use	53	34.86	
**Type of clothing usually used**			
Usual loose clothes (long sleeves ,pants) with dark color	42	27.63	
Long loose clothing with light coloring.	106	69.73	
Short sleeve/pants/shorts	3	1.97	
**Overall Scores**	152	100	**30.00 (±12.00)**
**Adjustment variables**	**Frequency (n)**	**Relative frequency (%)**	**Adjustement score**

**Non-modifiable**	**Skin Phototype**			
	Type V & VI	5	3.28	**0.25**
	Type III &IV	30	19.73	**0.5**
	Type II Type I	111	73.02	**0.75**
	Type III	5	3.28	**1**
	**Weather outside** (sunny/light cloudy)			**0.75**
	**Sun Exposure Score (SES)**	**152**	**100**	**15.75 (±8.25)**
	**Sun exposure categories**			
	Insufficient sun exposure (<7.5)	2	1.31	
	Moderate sun exposure (7.5-15)	63	41.44	
	Sufficient sun exposure (15-30)	84	55.26	
	High sun exposure (>30)	2	1.31	
