# Supplementary material for: Development and Validation of Vitamin D- Food Frequency Questionnaire for Moroccan Women of Reproductive Age: Use of the Sun Exposure Score and the Method of Triad’s Model
Source: Nutrients. 2023 Feb 4;15(4):796. doi: 10.3390/nu15040796 (PMC9967684; doi:10.3390/nu15040796)
Supplement: Supplementary file 1 [file nutrients-15-00796-s001.zip › Table S4.pdf]

Table S4. Comparison of Median serum 25-OHD according to sunlight exposure score levels

Sunlight exposure factors	Serum 25-OHD (ng/mL) median ± IQR	P-value [a]
Insufficient	3.89	<0.001
Moderate	6.61±4.78	
Suficient	10.48±6.87	
High	25.43	
**Pairwise comparison**[b]	8.48±6.55	
Insufficient vs moderate		0.12
Insufficient vs sufficient		0.03
Insufficient vs high		0.12
Moderate vs sufficient		<0.001
Moderate vs high		<0.001
Sufficient vs high		0.02

[a] Kruskal Wallis H test

[b]Post hoc Mann-Whitney U tests

IQR : interquartil range.
